# Teacher–Student Relationships and Coping Styles in Chinese Children: The Chain Mediating Role of Peer Relationships and Psychological *Suzhi*

**DOI:** 10.3390/bs14090797

**Published:** 2024-09-10

**Authors:** Xinyi Wang, Chunli Yao, Shuowei Su, Xin Yu, Ningxuan Bai, Shuang Gao

**Affiliations:** School of Health Management, Guangzhou Medical University, Guangzhou 511436, China

**Keywords:** teacher–student relationships, coping styles, peer relationships, psychological *suzhi*, children

## Abstract

As age increases, children will face more and more adversity. How effectively they cope with stress and difficulties of life is of great significance to the development of children’s mental health and academic achievement. However, few studies have explored how different interpersonal relationships and psychological *suzhi* work together to influence children’s healthy behaviors, particularly healthy coping in adversity. Therefore, this research focused on the teacher–student relationships and coping styles, as well as the chain-mediated effects of peer relationships and psychological *suzhi*. A total of 688 children (360 boys, 52.3%; Mage = 10.98 and *SD* = 0.89) completed questionnaires that assessed using teacher–student relationships, peer relationships, psychological *suzhi*, and coping styles. The results indicated that teacher–student relationships correlated positively with coping styles, peer relationships, and psychological *suzhi* in children. Besides, teacher–student relationships positively affected coping styles through both the mediating roles of peer relationships and psychological *suzhi*. This research elucidated the extrinsic and intrinsic factors impacting the coping styles of children, thus providing empirical validation of existing theoretical frameworks. In China, interventions aimed at promoting Chinese children’s positive coping could benefit from strategies focused on cultivating high-quality relationships and enhancing psychological *suzhi*.

## 1. Introduction

Teacher–student relationships refer to the psychological connections formed through cognitive, emotional, and behavioral interactions between educators and students [1]. Youth development was linked to benign interaction with teachers and students, encompassing improved emotional and behavioral adjustment [2], academic development [3,4], and subjective well-being [5]. It is worth mentioning that multiple studies have shown that negative teacher–student relationships might contribute to maladaptive behavior patterns, depression, distress, and even suicidal behaviors [6,7,8]. Based on previous literature, teacher–student relationships may favor children’s positive coping in the face of negative physical or psychological consequences.

Coping styles are a set of general strategies used to handle stressful situations when faced with adversity, demonstrated through behavioral and cognitive processes [9]. Different coping styles can be identified based on individual coping characteristics. For instance, coping consists of problem-focused coping (i.e., centered on changing the situation and solving the problem) and emotion-focused coping (i.e., centered on managing negative emotions related to the situation) [10]. Another way to categorize coping is by analyzing whether it is approach-oriented or avoidance-oriented. Specifically, approach-oriented coping involves dealing with reality objectively and diligently, while avoidance-oriented coping involves attempting to ignore stressful events and isolate feelings [11]. Individuals’ attitudes and behaviors in adversity are directly influenced by the type of coping styles they use, which highlights the significance of coping styles in personal growth and mental health. What is more, student development in educational settings is synthetically affected by coping styles. Research suggested that positive coping styles were associated with higher levels of learning motivation [12], academic resilience [13], emotion regulation, and subjective well-being [14] in student populations. However, negative coping styles were predictors of potential mental disorders, which increased the likelihood of maladaptive cognition and problematic behaviors among students [15]. A systematic review showed that the socio-emotional skills training programs for children focused on enhancing their stress management and building adaptive coping skills [16]. Researchers also highlighted the significance of teachers’ support and positive school climate, which helped children overcome academic challenges and deal with peer rejection, ultimately enhancing their problem-focused coping and adaptation [17].

China is a country with a strong emphasis on collectivism, where the importance of harmonious group interactions and the cultivation of interpersonal relationships is highly valued. Hence, in a collectivist culture, fostering good interpersonal relationships becomes crucial for enhancing mental health and facilitating effective coping styles [18,19]. A recent study discovered no clear link between negative coping styles and teacher–student relationships in adolescents [20]. This suggests that as adolescents become more self-aware, their negative coping styles may be more closely tied to internal factors [21]. However, children’s coping strategies could be easily influenced by the adults in their lives [22]. Previous studies have not adequately addressed whether coping styles and teacher–student relationships are influenced by age. We believe that this association cannot be universally applied to children. Past research has offered limited insights into how teacher–student relationships affect coping styles in children. The main purpose of this study was to explore the connection between teacher–student relationships and children’s coping styles, as well as the factors that may influence this correlation. The aim was to uncover the protective factors of children’s health behaviors, which can help mitigate negative coping strategies and enhance their mental well-being.

### 1.1. The Relationship between Teacher–Student Relationships and Coping Styles

The coping styles one adopts are determined by the type of events they are facing. In China, coping styles are classified into two categories: active coping (e.g., making positive changes) and passive coping (e.g., illusion and negative venting behaviors) [23]. Research has indicated that adults who use a coping style centered on avoiding reality are at a higher risk for alcohol and substance abuse and have increased levels of depression [24]. Research on children has found similar results regarding the correlation between coping styles and mental well-being. Specifically, negative coping styles were linked to anxiety symptoms and perceptions of chronic social adversity [25,26].

Drawing inspiration from the social bond theory, social relationships have a crucial effect on shaping healthy behaviors in individuals [27]. Research showed that positive teacher–student relationships may encourage students to build positive coping styles that develop effective strategies to deal with boredom in language learning [28]. The interactions between teachers and students can encourage students to cope with challenges actively in their school engagement and learning process [20]. Furthermore, fostering positive teacher–student relationships is essential for students to adapt to the school context and reduce student victimization in schools [29,30,31]. Effective teachers establish supportive and non-judgmental classroom environments, which can meet the psychological and behavioral needs of students with emotional or behavioral disorders and ultimately enhance their personal dispositions and abilities [32]. On the other hand, high-conflict teacher–student relationships put children at risk for adverse life events and negative outcomes, including behavioral disorders. In this sense, teacher–student relationships might positively influence coping styles in children.

**Hypothesis** **1.** *Teacher–student relationships significantly and positively predict coping styles in children*.

### 1.2. The Mediating Effect of Peer Relationships

Peer relationships refer to the interpersonal connections between individuals who are at a similar stage of psychological development [33]. As peer relationship research advances, there has been extensive exploration into the connection between peer relationships and psychopathology. Positive peer relationships are crucial for healthy child development, including benefits such as social support, exercise adherence, and academic achievement [34]. Conversely, negative peer relationships were linked to many adverse outcomes, including family victimization, mental illness, and involvement in criminal activities [35]. Research showed that overweight children were prone to negative peer relationships and mental health conditions due to peer problems. The correlation between overweight and mental health issues was largely affected by the existence of peer problems [36].

Based on self-determination theory, individuals tend to improve their mental well-being when the three inherent psychological needs, including relatedness, autonomy, and competence, are fulfilled [37]. Specifically, the fulfillment of relatedness is primarily demonstrated through the growth of interpersonal connections, specifically encompassing the bonds between parents and children, teachers and students, as well as peers. As a positive interpersonal factor, positive peer relationships might also promote children’s coping styles in communication. A recent systematic review has reported that peer relationships can positively influence the way young people cope [38]. Furthermore, prior research also showed that teacher–student relationships and parent–child interactions were positively correlated with student’s peer relationships [39,40]. This serves as a reminder that children’s interpersonal relationships with adults, such as teachers, might impact relationships with peers and the development of behavioral patterns [32]. In conclusion, the evidence presented above reinforces the idea proposed in this study that peer relationships might mediate the correlation between teacher–student relationships and children’s coping styles.

**Hypothesis** **2a.** *Teacher–student relationships significantly and positively predict peer relationships in children*.

**Hypothesis** **2b.** *Peer relationships significantly and positively predict coping styles in children*.

### 1.3. The Mediating Effect of Psychological Suzhi

Psychological *suzhi* is based on physiological factors, converting external stimuli into stable, implicit, and beneficial psychological qualities that are closely linked to adaptability and innovative behaviors [41]. The concept originated under the framework of quality education in China [42]. Moreover, psychological *suzhi* is impacted by both internal and external factors and highlights three main dimensions: cognitive quality, individuality, and adaptability [41]. Cognitive quality, in particular, has a crucial effect on the cognitive process, self-regulation, and constructive responses to challenges. The significance of psychological *suzhi* is highly emphasized in China, as it is considered crucial for both social harmony and personal success. Prior research suggested that the enhancement of children’s psychological *suzhi* was conducive to unlocking their potential, shaping character, molding personality, and ultimately enhancing their social adaptability [43]. In addition, studies indicated that psychological *suzhi* positively predicted Chinese children’s healthy behavior habits [44], classroom peer status [45], and academic achievement [46]. It is noteworthy that psychological *suzhi* is integrated into various educational practices and personal development programs [43]. Furthermore, the American *Handbook of Positive Psychology in Schools* emphasized the scientific research on psychological *suzhi* in China as a part of the research on positive psychology in Chinese schools within the framework of quality education. This also indicates that the international community is taking notice of the research on psychological *suzhi* in a positive way [47].

Stage–environment fit theory suggests that a supportive environment can encourage positive development [48]. For students, a supportive climate in school is also essential for healthy growth and development [49]. As one of the dimensions of perceived school climate, teacher–student relationships play an active role in mental health. Prior studies showed a positive relationship between teacher–student relationships and psychological *suzhi* [50,51]. Specifically, good teacher–student relationships might promote the construction of cognitive skills and adaptability among students, resulting in improved psychological *suzhi*. What is more, individuals apply different coping styles when faced with stressful situations, leading to shifts in physiological, psychological, and behavioral reactions. Psychological *suzhi*, as a stable and inherent psychological quality, is advantageous for individuals in coping with challenges and hardships. Prior research showed that male adolescents with high psychological *suzhi* exhibited reduced state anxiety and heart rate responses during the Trier Social Stress Test [52], suggesting that psychological *suzhi* was a critical factor that encouraged individuals to adopt active coping styles when confronted with acute stress. Furthermore, relevant research also showed that psychological *suzhi* may be related to the construction of effective coping strategies. Firstly, it serves as a protective shield that weaken the impact of stressful life situations on sleep quality, allowing students to better navigate through stressful situations [53]. Secondly, psychological *suzhi* acts as a safeguard against social anxiety, which may improve the social function of students through its influence on self-esteem and sense of security [54].

**Hypothesis** **3a.** *Teacher–student relationships significantly and positively predict psychological suzhi in children*.

**Hypothesis** **3b.** *Psychological suzhi significantly and positively predicts coping styles in children*.

### 1.4. The Chain Mediating Effect of Peer Relationships and Psychological Suzhi

The quality of interpersonal relations is closely related to an individual’s behavior patterns and psychological characteristics. As one of the critical social relations, peer relationships can affect student’s mental health. High-quality friendships among peers can provide emotional support, competence, and enjoyable social interactions [55]. Conversely, peer rejection led children to report more negative self-perceptions, hindering their adaptive development [55,56]. Negative peer relationships, such as peer victimization, could also result in children and their parents reporting more symptoms of psychopathology, increasing their risk of mental health problems [57]. Prior studies have shown that peer relationships are positively associated with psychological *suzhi* [58,59]. Specifically, engaging in cooperative activities with peers can help children feel more competent in schoolwork and a sense of belonging, leading to positive self-evaluations of their behaviors. Children with good peer relationships tend to have more psychological resources and satisfaction than those with negative peer relationships, thus enriching their psychological *suzhi*. Therefore, peer relationships and psychological *suzhi* might have chain-mediated effects on the relationship between teacher–student relationships and coping styles.

**Hypothesis** **4.** *Peer relationships significantly and positively predict psychological suzhi in children*.

### 1.5. The Present Research

In the field of child development research, interpersonal relationships of children have been a crucial factor in social adjustment and mental health. Despite extensive exploration, most existing research has focused primarily on positive developmental outcomes related to children’s single interpersonal relationships, such as academic achievement. However, there is a notable lack of research on the interactions between different interpersonal relationships in children’s specific environments, particularly with regard to teacher–student relationships. Considering the critical effect of coping styles in facing adversity, the present research aims to fill this gap by investigating the specific mechanisms between teacher–student relationships and children’s coping styles in the school contexts, exploring the chain-mediated effects of peer relationships and psychological *suzhi*.

**Figure 1:**
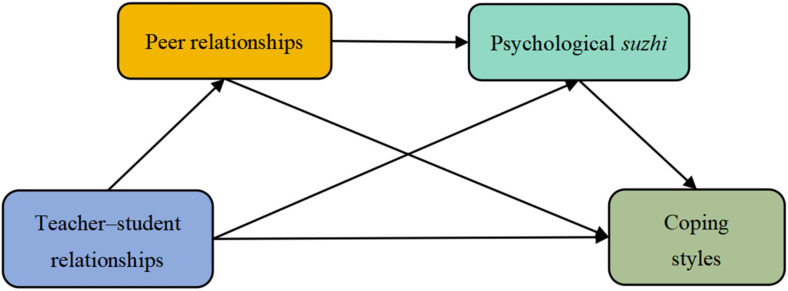
The assumed model of the chain mediating role of peer relationships and psychological *suzhi* in the relationship between teacher–student relationships and coping styles.

In summary, based on the social bond theory, self-determination theory, stage-environment fit theory, and previous studies, there is a close correlation between teacher–student relationships, peer relationships, psychological *suzhi*, and coping styles. While previous research has shown connections between the four core variables, there is a lack of studies that specifically create a chain mediation model involving these variables in children samples. Through the construction of the model, we aim to delve into the potential mechanism through which teacher–student relationships impact children’s coping styles. This research will offer a theoretical foundation for enhancing and intervening in children’s coping styles. The chain mediation model is depicted in Figure 1.

## 2. Methods

### 2.1. Procedures

The data were obtained from a stratified sample of 758 fourth-, fifth-, and sixth-grade children in China (Guangdong Province). The selection criteria included ensuring that the children did not have any psychiatric or neurological disorders, as reported by their teachers in schools. All participants gave informed consent before completing the questionnaires, indicating their voluntary participation and understanding of the purpose, expected duration, and procedures. This study was carried out in compliance with the Declaration of Helsinki [60]: we protected the participants’ rights in the research process. The informed consent form we offered ensured that the conventional ethical procedures were clear, including that participants’ information would be kept confidential and the anonymity of reporting was guaranteed. Besides, we followed the ethical guidelines of the American Psychological Association (APA) [61], making sure that researchers in this study program were in good integrity, justice, and responsibility.

Questionnaires were handed out by teachers, and participants were required to complete the demographic information with paper and pen in 25 min, along with the following questionnaires: (1) The Simplified Coping Style Scale [23]; (2) Teacher–Student Relationship Scale and Classmate Relationship Scale [62]; and (3) Simplified Version of the Psychological *Suzhi* Scale [63]. Participants completed the self-report questionnaires in a quiet classroom within 25 min. The research protocol received approval from the medical ethics committee at Guangzhou Medical University. After excluding 70 samples that did not meet the statistical criteria and had missing data, we proceeded with the data analysis, with an effective recovery rate of 90.8%.

### 2.2. Participants

The final participant pool comprised 360 boys (52.3%), with an average age of 10.95 years (*SD* = 0.94), and 328 girls (47.7%), with an average age of 11.02 years (*SD* = 0.83). This sample included 236 fourth-grade students, 221 fifth-grade students, and 231 sixth-grade students. In addition, there were 153 only children and 535 non-only children. Further exploring the demographic profile in this study, we found a predominance of non-only children in the sample, which may be attributed to the relaxation of China’s birth policies since 2013. It is of concern that China’s only children reported lower levels of psychological distress compared to their peers with siblings [64]. Hence, this variable might be a significant factor influencing children’s mental development, and our research would also focus on this variable.

### 2.3. Measures

#### 2.3.1. Coping Styles

The Simplified Coping Style Scale was used to measure coping styles in our study [23]. This scale consists of 20 items on a four-point Likert scale ranging from 0 (never adopted) to 3 (often adopted). The scale is split into two subscales: positive and negative coping styles. The positive coping style subscale mainly measures features of active coping, such as “talking to someone about inner troubles” and “asking someone for possible advice” when experiencing adversity. The negative coping style subscale measures features of passive coping, including “escaping difficulties” and “waiting without purpose or significance” when facing adversity. The coping tendency score is calculated by subtracting the mean of negative coping styles from the mean of positive coping styles, with a score above 0 indicating a tendency toward active coping and a score below 0 indicating a tendency toward passive coping. In this study, the Cronbach’s alpha coefficient was 0.904, the measurement model fit indices were as follows: χ^2^/df = 5.100, RMSEA = 0.077, SRMR = 0.012, CFI = 0.996, and TLI = 0.982, and the scale had good structural validity.

#### 2.3.2. Teacher–Student Relationships

The Teacher–Student Relationship Scale was a subscale of the “My Class Scale”, which was a well-established scale in China [62]. This scale is composed of eight items and uses a five-point Likert format, ranging from 1 (strongly disagree) to 5 (strongly agree). The instrument is designed to measure the quality of the relationship between teachers and students. It includes items like “classroom teacher cares about classmates” and “the classroom teacher takes into account the self-esteem of students”, focusing on the interactions between teachers and students. Participants with higher scale scores reported better teacher–student relationships. In this study, the scale demonstrated a Cronbach’s alpha coefficient of 0.927; the measurement model fit indices were as follows: χ^2^/df = 3.442, RMSEA = 0.060, SRMR = 0.020, CFI = 0.991, and TLI = 0.979, and this scale had good structural validity.

#### 2.3.3. Peer Relationships

Peer relationship was measured by the Classmate Relationship Scale for elementary school students [62]. This scale is composed of eight items and uses a five-point Likert format, ranging from 1 (strongly disagree) to 5 (strongly agree). The instrument is designed to measure the quality of peer relationships. It includes items like “classmates provide support and encouragement to one another” and “peers can speak truthfully to others”, focusing on the interactions with peers. Participants with higher scale scores reported better peer relationships. In this study, the scale demonstrated a Cronbach’s alpha coefficient of 0.769; the measurement model fit indices were as follows: χ^2^/df = 2.798, RMSEA = 0.051, SRMR = 0.031, CFI = 0.979, and TLI = 0.966, and this scale had good structural validity.

#### 2.3.4. Psychological *Suzhi*

Psychological *suzhi* was assessed using the simplified version of the Psychological *Suzhi* Scale. The scale was created to measure positive psychological characteristics, which contribute to the successful adaptation of Chinese students to the school context [63]. This scale is composed of 27 items and uses a five-point Likert format, ranging from 1 (strongly disagree) to 5 (strongly agree). The scale is split into three subscales: cognitive quality, personality quality, and adaptability. Among them, cognitive quality includes metacognitive awareness, planning, and monitoring; personality quality includes confidence, self-esteem, responsibility, and optimism; adaptability includes emotional adaptation, interpersonal adaptation, school adaptation, and frustration tolerance. Each dimension has nine items. Higher scores on the scale indicate higher levels of psychological *suzhi*. In this study, the scale demonstrated a Cronbach’s alpha coefficient of 0.945; the measurement model fit indices were as follows: χ^2^/df = 3.421, RMSEA = 0.059, SRMR = 0.016, CFI = 0.992, and TLI = 0.981, and this scale had good structural validity.

### 2.4. Data Analysis

We utilized IBM SPSS 26.0 and Amos 26.0 software to analyze data: (1) the scale’s reliability and validity in this study were assessed using Cronbach’s coefficients and the fitted model indicators; (2) we computed means, standard deviations, and Pearson’s correlation coefficients for assessing the levels of teacher–student relationships, coping styles, peer relationships, and psychological *suzhi* in Chinese children; (3) the variables of gender, age, and only child status were analyzed in this study, as it was reported that gender, age, and only child status were related to the relationships and coping styles of individuals [65,66,67]; (4) the data was standardized using the Z standardization method in SPSS 26.0 software, which involved standardizing each variable by subtracting the mean and dividing by the standard deviation. In this study, the mediation effect was analyzed using Model 6 in the SPSS PROCESS macro version 3.3 created by Hayes [68]. First, regression analyses explored the relationships between each variable, as well as the association indices in the analyses. Besides, the significance of the mediation effect was tested using the bias-corrected percentile bootstrap method. Harman’s single-factor test can reduce the bias effect caused by the common method [69]. The findings revealed that nine factors had eigenvalues exceeding 1, with the first factor explaining 30.69% of the variance, falling below the 40% critical value criterion. It suggested that no significant common method bias was detected in this study.

## 3. Results

### 3.1. Descriptive Statistics, Correlation Analysis, and Analysis of Variance of Variables

Descriptive analyses showed that the mean value of the scores of coping styles was greater than 0, which indicated that the investigated group of children tended to use positive coping styles. Correlation analyses showed that teacher–student relationships were positively correlated with coping styles, peer relationships, and psychological *suzhi* (all *p* < 0.01), with correlation coefficients *r* ranging from 0.423 to 0.655. Besides, significant positive correlations were noted between the three key variables of coping styles, peer relationships, and psychological *suzhi* (all *p* < 0.01), with correlation coefficients *r* ranging from 0.362 to 0.551. The correlation analysis provided support for testing the hypothesis model and conducting mediation analysis (see Table 1).

To explore the potential impact of demographic factors (gender, grade, only child status) on teacher–student relationships, peer relationships, psychological *suzhi*, and coping styles, we performed independent samples *t*-tests and ANOVA (see Table 2). Our findings revealed no significant difference between children based on gender, grade, and only child status in terms of teacher–student relationships, peer relationships, and coping styles (all *p* > 0.05). This suggested that gender, grade, and only child status did not have a differential impact on teacher–student relationships, peer relationships, and coping styles in children. However, we did observe a statistically significant difference (all *p* < 0.05) in psychological *suzhi* scores between boys and girls, as well as between only children and non-only children. This indicated that gender and only child status might have impacts on psychological *suzhi* in children, providing a basis for further regression analysis.

### 3.2. Examining the Mediation Model

**Figure 2:**
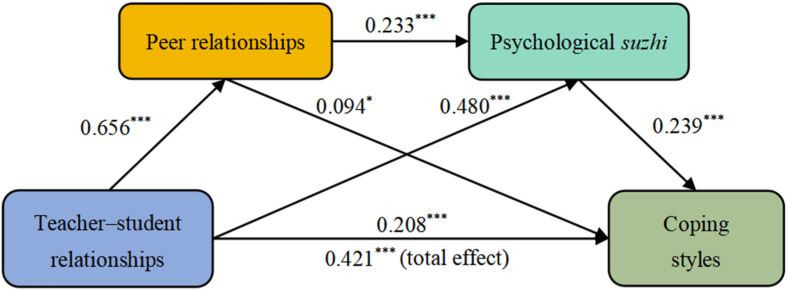
A mediating model of teacher–student relationships affecting coping styles. * *p* < 0.05, *** *p* < 0.001.

In this study, we utilized the PROCESS macro version 3.3 for SPSS and selected model 6 to analyze the data. The study investigated the impact of teacher–student relationships on children’s coping styles and considered the mediating effects of peer relationships and psychological *suzhi*, while controlling for gender, grade, and only child status. The findings of regression analyses (see Table 3 and Figure 2) revealed that teacher–student relationships positively predicted coping styles (*β* = 0.421, *t* = 12.144, *p* < 0.001), which showed the total effect of teacher–student relationships on coping styles. Secondly, teacher–student relationships positively predicted peer relationships (*β* = 0.656, *t* = 22.713, *p* < 0.001). Besides, teacher–student relationships (*β* = 0.480, *t* = 12.602, *p* < 0.001) and peer relationships (*β* = 0.233, *t* = 6.114, *p* < 0.001) positively predicted psychological *suzhi*. Lastly, when all variables were considered together, the effects of three variables on coping styles could be observed simultaneously. The result showed that teacher–student relationships significantly predicted children’s coping styles (*β* = 0.208, *t* = 4.192, *p* < 0.001), which showed the direct effect of teacher–student relationships on coping styles. Moreover, peer relationships (*β* = 0.094, *t* = 2.052, *p* < 0.05) and psychological *suzhi* (*β* = 0.239, *t* = 5.309, *p* < 0.001) also significantly predicted coping styles. In this regression analysis, the coefficient of determination (*R*^2^) was 0.227. Therefore, Hypotheses 1, 2a, 2b, 3a, 3b, and 4 of this study were confirmed.

We tested a mediation model using the percentile Bootstrap method (repeated sampling 5000 times) to gain a better understanding of the chain-mediated effects of peer relationships and psychological *suzhi* in our study (see Table 4). If the 95% confidence interval does not encompass zero, the impact of this pathway is considered significant. The results of the total indirect effects of peer relationships and psychological *suzhi* revealed that the 95% confidence intervals did not encompass zero (BootLLCI = 0.140, BootULCI = 0.287). This indicated that peer relationships and psychological *suzhi* played a mediating role in the relationships between teacher–student relationships and coping styles, with a total indirect effect size of 0.213. Further analyses indicated that the total indirect effect was composed of three mediating pathways: (1) teacher–student relationships → peer relationships → coping styles, with 95% confidence intervals for this pathway that did not encompass zero (BootLLCI = 0.002, BootULCI = 0.120), manifesting a significant indirect effect with an effect size of 0.062; (2) teacher–student relationships → psychological *suzhi* → coping styles, with 95% confidence interval for this pathway that did not encompass zero (BootLLCI = 0.070, BootULCI = 0.164), manifesting a significant indirect effect with an effect size of 0.115; (3) teacher–student relationships → peer relationships → psychological *suzhi* → coping styles, with 95% confidence interval for this pathway that did not encompass zero (BootLLCI = 0.018, BootULCI = 0.060), manifesting a significant indirect effect with an effect size of 0.036. Moreover, the 95% confidence intervals for the direct effects of teacher–student relationships and coping styles did not encompass zero (BootLLCI = 0.111, BootULCI = 0.306), and was with a direct effect size of 0.208. In terms of effect size, the chain-mediated effect in this study was weak. The 95% confidence intervals for the aggregate effect did not encompass zero (BootLLCI = 0.353, BootULCI = 0.489), and the aggregate effect size was 0.421. In conclusion, the results showed that teacher–student relationships could positively affect children’s coping styles. Peer relationships and psychological *suzhi* partially mediated the effects of teacher–student relationships on children’s coping styles. What is more, peer relationships and psychological *suzhi* had chain mediating effects of teacher–student relationships and coping styles in children.

## 4. Discussion

To explore the intrinsic mechanism of teacher–student relationships affecting children’s coping styles, this study constructed a chain mediation model mediated by peer relationships and psychological *suzhi*. Moreover, the study also provided an empirical basis for improving children’s coping styles and promoting their mental health and future development.

The results of our study show that teacher–student relationships have a significant and positive effect on children’s coping styles, which confirms Hypothesis 1 of this study. Research has demonstrated that coping styles have beneficial impacts on alleviating mental health symptoms [70]. Conversely, negative coping styles have been identified as a significant factor in mental disorders, such as depression. This highlights the importance of providing behavioral and mental health support to school-aged children and adolescents. Positive teacher–student relationships might improve attachment relationship quality and objective self-perceptions [31], while perceived conflict, harassment, and other negative aspects of teacher–student relationships can contribute to heightened emotional and behavioral problems [71,72]. Moreover, when there is a negative relationship between teachers and students, such as conflicts between them, it can lead to an improved risk of depression. It is of concern that the negative impact of teacher–student conflict outweighed the benefits of teacher–student warmth in this relationship [73]. Recent research has suggested that negative teacher–student interactions can lead to strained relationships and more oppositional behaviors [74]. Therefore, we believe that reducing teacher–student conflict needs to be given special attention in cultivating positive teacher–student relationships, which may reduce the occurrence of children’s negative feelings and bad behaviors. The results showed that Chinese children tended to adopt positive coping styles. Our study supports previous findings that strong teacher–student relationships not only benefit children in shaping healthier psychological and behavioral patterns but also promote adaptive coping when facing adversity in the school context, especially the positive coping styles of children.

The study results indicate that teacher–student relationships positively predict peer relationships, and, in turn, peer relationships positively predict coping styles; peer relationships have a mediating role in the relationships between teacher–student relationships and children’s coping styles. This finding confirms Hypotheses 2a and 2b of this study. One potential explanation for this finding is that positive teacher–student relationships tend to facilitate effective attainment of greater peer liking and acceptance, which may also strengthen children’s psychological *suzhi* [75]. From another perspective, children may also be more inclined to form friendships with peers who have good relationships with their teachers, reflecting the characteristics of developing peer relationships among children. Children who perceived harmonious teacher–student relationships tended to report more prosocial behaviors, which allowed them to better integrate into student groups [76]. On the contrary, studies have found that adolescents in severely antisocial peer groups tended to have more risky behaviors [77]. Negative peer relationships were also associated with substance use as well as externalizing symptoms in adolescents [78]. In addition, self-determination theory emphasizes that the satisfaction of relatedness can improve mental health and decrease the risk of mental illness. While research has demonstrated the significance of relationships with parents, teachers, and peers, surprisingly limited research has explored the collective impact of these relationships [32,33]. This study suggests that teacher–student relationships may promote positive coping with adversity in children through peer relationships, thus establishing positive coping styles. We provide empirical support for the link between the need for relatedness and personal development in self-determination theory, clarifying the importance of relationships with teachers and peers in influencing children’s coping styles.

Moreover, in the Chinese cultural context, the study results indicate that teacher–student relationships positively predict psychological *suzhi*, and, in turn, psychological *suzhi* positively predict coping styles; psychological *suzhi* has a mediating role in the relationships between teacher–student relationships and children’s coping styles. This finding confirms Hypotheses 3a and 3b of this study. A previous study has shown that the relationship between teachers and students is a significant factor in determining the level of closeness, social interactions, and academic achievement [79]. Teachers play a crucial role in providing social support to children, which, in turn, has a beneficial effect on their mental well-being and future growth. As an endogenous factor for mental health, psychological *suzhi* also plays a critical role in shaping health behaviors and reducing negative behaviors in children [80,81]. Correspondingly, children who received teacher support are more inclined to have benign interaction with the teacher, and it may improve their psychological *suzhi*. Due to the improvement of psychological *suzhi*, children might tend to build a more positive coping style. Besides, we found that boys have higher psychological *suzhi* than girls, while the psychological *suzhi* of non-only children is higher than that of only children. This may be due to the fact that boys have internalized the idea of masculinity and express higher confidence [82], which will improve their personality quality. Moreover, different family compositions and sizes shape various family interaction patterns. For non-only children, the presence of siblings provides them with more opportunities to interact with peers and demonstrates higher agreeableness [83], which may help enhance their adaptability.

The results also indicate that peer relationships have a significant and positive effect on psychological *suzhi*, which confirms Hypothesis 4 of this study. Moreover, the connection between teacher–student relationships and coping styles is sequentially mediated by peer relationships and psychological *suzhi*. When children perceive harmonious teacher–student relationships, they might develop good interpersonal relationships and inner qualities [84]. Besides, Pan and colleagues [59] found that peer attachment has a notable and favorable impact on psychological *suzhi*. Specifically, children with negative peer relationships may have lower peer liking and psychological *suzhi* than those with positive peer relationships. Thus, fostering an inclusive peer environment might facilitate children’s peer experience and psychological *suzhi*, thereby encouraging positive coping styles. This indicates that when teacher–student relationships lack warmth, school leaders can introduce initiatives to improve these relationships, which will elevate the school’s education quality [85].

## 5. Implications, Limitations, and Future Research Directions

The results of the study have theoretical significance and practical value for promoting coping styles in children. From a theoretical perspective, the findings suggest that teachers have a multifaceted influence on children. In addition to their role in education, teachers also play a critical part in encouraging children’s emotional, behavioral, and social development. By building good relationships with students and creating a supportive environment, teachers contribute to children’s psychological *suzhi* and facilitate them to form healthy peer relationships and adaptive coping styles. In turn, these positive qualities also contribute to children’s resilience and success in social, academic, and mental aspects.

Moreover, to enhance children’s coping styles, the following suggestions are proposed: First, the formation of coping styles is crucial during childhood. Thus, attention should be paid to children who perceive the teacher–student relationships as negative, which may imply that their coping styles are more negative than those of their peers and report more psychopathology symptoms. Second, in designing programs to improve positive coping, the significance of social relationships must be emphasized, and this includes fostering teacher–student and peer relationships. When interviewing children with negative coping styles, school counselors can encourage these children to participate in classroom activities, which may help them gain positive interpersonal relationships from these activities. Moreover, psychology teachers can organize and conduct consistent psychological *suzhi* training sessions to boost children’s psychological *suzhi*, potentially leading to an enhancement in their coping styles. Finally, psychological training can be carried out in schools, which aims to teach children to cultivate adaptive coping styles and resilience and enhance the ability to address problems, thereby improving their mental health.

The findings hold great importance for programs designed to improve children’s coping styles. While there are some highlights, the present study had several limitations that required further interpretation. First, due to the cross-sectional design of our research, we were unable to establish a causal relationship between the study variables. Second, although this study found that the chain-mediated pathway of peer relationships and psychological *suzhi* is significant, the mediating effects and the chain mediating effect were comparatively weak in the study. Understood in terms of regression analysis, the results of the coefficient of determination also reflect the presence of other important variables in the impact of teacher–student relationships on children’s coping styles [86]. Third, because all data are based on self-reporting by children, our results may be affected by biases due to social desirability. This study included only Chinese children; generalizing the findings to other cultures or age groups requires caution. Therefore, future experimental and longitudinal studies should further examine the findings of this study by delving more deeply into the mechanisms of influence among the variables investigated. Moreover, we will also explore the critical factors involved between teacher–student relationships and children’s coping styles, which are worth further exploration in future research. Although no deviation from common methods was observed, it is recommended that research utilizes different types of data collection methodologies in the future, such as combining self-reports with reports from others (e.g., teachers and parents), to enhance the reliability of the conclusions. Further studies can investigate how interpersonal relationships impact children’s coping styles in different groups and cultural contexts.

## 6. Conclusions

This study investigated how teacher–student relationships impact children’s coping styles, specifically looking at how peer relationships and psychological *suzhi* mediate this relationship. Using a chain mediation model, the study reached the following conclusions: (1) Teacher–student relationships, as extrinsic factors, can positively predict children’s coping styles, peer relationships, and psychological *suzhi*. (2) Peer relationships, as extrinsic factors, can positively impact children’s coping styles and psychological *suzhi*. (3) Psychological *suzhi*, as an intrinsic quality, also plays a positive role in shaping coping styles in children. (4) Peer relationships and psychological *suzhi* play mediating roles in the link between teacher–student relationships and children’s coping styles, operating through three different pathways: the mediation of peer relationships, the mediation of psychological *suzhi*, and the chain mediation of peer relationships and psychological *suzhi*.

## Figures and Tables

**Table 1:** Pearson correlation coefficient.

	M	SD	1	2	3	4
1. Teacher–student relationships	2.55	0.76	1			
2. Peer relationships	2.72	1.01	0.655 **	1		
3. Psychological *suzhi*	3.68	0.79	0.634 **	0.551 **	1	
4. Coping styles	0.37	0.66	0.423 **	0.362 **	0.423 **	1

** *p* < 0.01.

**Table 2:** Scale scores for different demographic characteristics of 688 students.

Project	Number	Teacher–Student Relationships	Peer Relationships	Psychological *Suzhi*	Coping Styles
Gender					
Boy	360	2.57 ± 0.74	2.78 ± 0.95	3.74 ± 0.80	0.38 ± 0.68
Girl	328	2.52 ± 0.78	2.66 ± 1.08	3.62 ± 0.77	0.36 ± 0.65
*t*		0.938	1.526	1.999	0.347
*p*		>0.05	>0.05	<0.05	>0.05
Grade					
Grade 4	236	2.52 ± 0.75	2.71 ± 0.97	3.70 ± 0.75	0.36 ± 0.64
Grade 5	221	2.52 ± 0.70	2.79 ± 1.03	3.63 ± 0.81	0.30 ± 0.69
Grade 6	231	2.60 ± 0.82	2.67 ± 1.05	3.71 ± 0.80	0.44 ± 0.65
*F*		0.968	0.743	0.780	2.474
*p*		>0.05	>0.05	>0.05	>0.05
Only child status					
Yes	153	2.54 ± 0.83	2.68 ± 1.12	3.60 ± 0.88	0.32 ± 0.72
No	535	2.55 ± 0.74	2.73 ± 0.98	3.70 ± 0.76	0.38 ± 0.65
*t*		−0.199	−0.535	−1.357	−1.131
*p*		>0.05	>0.05	<0.05	>0.05

**Table 3:** Regression analyses of chain mediation effects of peer relationships and psychological *suzhi*.

Regression Equation		Overall Fit Index			Significance of Regression Coefficients	
Outcome Variable	Predictor Variable	*R*	*R* ^2^	*F*	*β*	*t*
Coping styles	Teacher–student relationships (total effect)	0.426	0.182	37.956	0.421	12.144 ***
Peer relationships	Teacher–student relationships	0.658	0.433	130.248	0.656	22.713 ***
Psychological *suzhi*	Teacher–student relationships	0.662	0.439	106.674	0.480	12.602 ***
	Peer relationships				0.233	6.114 ***
Coping styles	Teacher–student relationships (direct effect)	0.476	0.227	33.259	0.208	4.192 ***
	Peer relationships				0.094	2.052 *
	Psychological *suzhi*				0.239	5.309 ***

* *p* <0.05; *** *p* <0.001.

**Table 4:** Bias-corrected bootstrap test on mediating effects.

Effect	Pathways	Effect	SE	BootLLCI	BootULCL
Direct effect	TSR → CS	0.208	0.050	0.111	0.306
Indirect effect	TSR → PR → CS	0.062	0.030	0.002	0.120
	TSR → PS → CS	0.115	0.024	0.070	0.164
	TSR → PR → PS → CS	0.036	0.011	0.018	0.060
Total indirect effect		0.213	0.037	0.140	0.287
Aggregate effect		0.421	0.035	0.353	0.489

TSR = teacher–student relationships; CS = coping styles; PR = peer relationships; PS = psychological *suzhi*.

## Data Availability

The original contributions presented in this study are included in the article. Further inquiries can be directed to the corresponding author.
